# Development of Gluten‐Free Biscuits From Rice Flour‐Pineapple Stem Starch Blends: Physicochemical Properties, In Vitro Starch Digestibility, Estimated Glycemic Index, Sensory Evaluation, and Storage Stability

**DOI:** 10.1002/fsn3.72449

**Published:** 2026-10-05

**Authors:** Jiratthitikan Sriprablom, Phenphop Phansuea, Yuraporn Sahasakul, Sasapin Disnil, Amornrat Aursalung, Nuttarat Srisangwan, Arisa Keeratichamroen, Supatcha Srisungwan, Rungtiwa Wongsagonsup

**Affiliations:** ^1^ Institute of Nutrition Mahidol University Nakhon Pathom Thailand

**Keywords:** estimated glycemic index, functional food, gluten‐free biscuits, pineapple stem starch, starch digestibility, storage stability

## Abstract

Pineapple stem starch (PSS) is a valuable source of nutritional resistant starch (RS), offering great potential as an alternative ingredient for producing functional gluten‐free biscuits. This study aimed to develop healthy gluten‐free biscuits using blends of all‐purpose rice flour (RF) and PSS and to evaluate their physicochemical properties, in vitro starch digestibility, estimated glycemic index (eGI), sensory attributes, and storage stability. The biscuits were prepared by combining RF and PSS at various ratios of 25:75, 50:50, 75:25, and 0:100, in comparison to gluten‐free control biscuits made with 100% RF. As the PSS content increased, the biscuit thickness increased, whereas hardness decreased. In terms of starch digestibility, the RS content of biscuits increased but eGI decreased with increasing PSS incorporation. Sensory evaluation revealed that biscuits containing 75% PSS received the highest liking scores across all assessed parameters. This optimum formulation (25:75 RF‐to‐PSS ratio) had an eGI of 60.04, which falls within the medium‐GI category. In addition, both the control biscuits and the 75% PSS biscuits maintained peroxide value (PV) and thiobarbituric acid reactive substances (TBARS) within the acceptable limits throughout the storage period. The findings demonstrate that PSS is a promising functional ingredient for gluten‐free biscuits. Incorporating 75% PSS and 25% RF into the biscuit formula effectively produced reduced‐GI gluten‐free biscuits, demonstrating their potential as a functional food.

## Graphical abstract

Pineapple stem starch (PSS) was incorporated into gluten‐free rice biscuits at 0%, 25%, 50%, 75%, and 100% substitution levels to enhance nutritional value by increasing resistant starch (RS) content and reducing estimated glycemic index (eGI). The 75% PSS formulation was identified as optimal, exhibiting the highest liking scores and a medium eGI (60.04). Both control and 75% PSS biscuits maintained peroxide values (PV) within acceptable limits throughout storage.

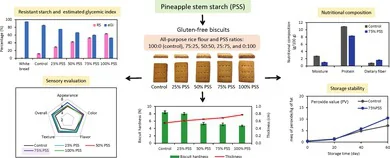

**Abbreviations**
AAS: Atomic absorption spectrophotometry
ANOVA: Analysis of variance
Cd: Cadmium
CMC: Carboxymethyl cellulose
eGI: Estimated glycemic index
GOPOD: Glucose oxidase/peroxidase
HI: Hydrolysis index
*k*
: Kinetic constant
MFS: Monk fruit sweetener
Pb: Lead
PSS: Pineapple stem starch
PV: Peroxide value
RDS: Rapidly digestible starch
RF: Rice flour
RS: Resistant starch
SDS: Slowly digestible starch
SPI: Soy protein isolate
SRBO: Shortening rice bran oil
TBARS: Thiobarbituric acid reactive substances
TS: Total starch

## 1 Introduction

The rising interest in developing healthier food products has led to extensive research and innovation in the field of food science and nutrition. In recent years, the use of alternative ingredients and fortification strategies has gained significant attention as a means of enhancing the nutritional value of conventional food items. Among these strategies, the incorporation of resistant starch as an alternative source of dietary fiber (Fuentes‐Zaragoza et al. 2010) into baked goods such as cookies, biscuits, and cakes (Cervini et al. 2021; Ceylan et al. 2021; Giuberti et al. 2017; Kahraman et al. 2019; Magallanes‐Cruz et al. 2020) has emerged as a promising avenue for producing healthier and more functional food products.

Biscuits are a popular snack enjoyed worldwide for their convenience, flavor variety, and long shelf life (Kahraman et al. 2019). However, biscuits made with wheat flour are unsuitable for individuals with gluten intolerance and celiac disease. As a result, to address this health concern, researchers have focused on creating gluten‐free biscuits or biscuits using alternative flour or starches, either native or modified forms such as rice, corn, gram, sorghum, millets, tapioca, and potato (Ali et al. 2023; Cervini et al. 2021; Giuberti et al. 2017; Putri et al. 2023; Schober et al. 2003; Sharma et al. 2016; Sresatan et al. 2024). These formulations are designed to enhance the nutritional profile, physical and sensory properties of gluten‐free biscuits. Recently, the enrichment of gluten‐free products with starches or flours containing resistant starch has gained increasing interest. Previous studies have revealed that the use of various sources of resistant starch, e.g., unripe banana flour, underexploited flours (from buckwheat, millet, sorghum, and legumes), annealed sorghum starch, and modified waxy rice starch, can effectively increase the resistant starch content and lower the glycemic index (GI) value of gluten‐free biscuits or cookies (Cairano et al. 2021; Cervini et al. 2021; Detchewa et al. 2021; Giuberti et al. 2017).

Pineapple stems, often considered agricultural waste from pineapple farms after fruit harvesting, hold significant potential as a valuable source of starch with distinct physicochemical properties (Nakthong et al. 2017; Rinju and Harikumaran‐Thampi 2021). Our previous study found that pineapple stem starch (PSS) has the highest levels of slowly digestible starch (SDS) and resistant starch (RS), which are related to its high amylose content (30.82%) compared to commercial rice, corn, and cassava starches (Sriprablom et al. 2023). SDS and RS are beneficial carbohydrates known to regulate blood glucose homeostasis and reduce glycemic response, potentially helping to prevent chronic conditions such as diabetes and obesity (Bello‐Perez et al. 2020; Fuentes‐Zaragoza et al. 2010). These unique properties make PSS a promising sustainable candidate for the development of functional foods with potential health benefits. In addition, this can simultaneously solve the problem of agricultural waste reduction from pineapple cultivation. In our previous studies, PSS was used to produce rice flour‐PSS noodles (Pinyo et al. 2024) and extruded products (Nisitthichai et al. 2024; Tangsrianugul et al. 2023). To the best of our knowledge, the scientific literature has not yet explored the use of PSS as an alternative source of RS for producing gluten‐free biscuits with a reduced estimated GI (eGI).

Therefore, this study aimed to develop healthy gluten‐free biscuits made from rice flour (RF) and PSS in various ratios. However, since both RF and PSS lack gluten proteins, this negatively impacts the texture and structure of gluten‐free biscuits. Therefore, other ingredients such as soy protein isolate (SPI) and carboxymethyl cellulose (CMC) could be added to enhance the functional and physical properties of gluten‐free biscuits (Nammakuna et al. 2009, 2016; Ogunbusola et al. 2020). In addition, these delicious treats can be made healthier by replacing butter or margarine and sugar with shortening rice bran oil (SRBO) and monk fruit sweetener (MFS), respectively. Replacing butter with SRBO reduces saturated fat while increasing essential unsaturated fatty acids (Kaur et al. 2012). MFS, a natural, calorie‐free sweetener, offers various health benefits, for exampl, regulating immune function and lowering glucose levels (Buchilina and Aryana 2020). In this study, the physical properties, nutritional composition, sensory attributes, and storage stability of the gluten‐free biscuits enriched with PSS were investigated. Furthermore, the potential health benefits of this fortification were assessed by evaluating the in vitro digestibility of starch in the developed biscuits. By examining the physiological impact of consuming these biscuits, their potential as a viable vehicle for delivering the beneficial RS along with a lower eGI was evaluated.

## 2 Materials and Methods

### 2.1 Materials and Chemicals

Pineapple stems ( *Ananas comosus* , Smooth Cayenne variety) were collected from mature pineapple plants at the postharvest stage, following fruit harvesting, from a pineapple farm in Kanchanaburi, Thailand, in August 2024. White‐colored all‐purpose rice flour (RF) was obtained from Cho Heng Rice Vermicelli Factory Co. Ltd. (Nakhon Pathom, Thailand). Other ingredients for biscuit making, that is, shortening rice bran oil (SRBO), monk fruit sweetener (MFS), salt, baking powder, sodium bicarbonate, soy protein isolate (SPI), carboxymethyl cellulose (CMC), egg, and vanilla butter flavor were purchased from the supermarket (Nakhon Pathom, Thailand). Pepsin (P7000, 250 units/mg), porcine pancreatin (P7545, 8 × USP specifications) and amyloglucosidase (A7095, 300 U/mL) were purchased from Sigma‐Aldrich Chemical Co. (St. Louis, MO, USA). Total starch assay kit and glucose oxidase/peroxidase (GOPOD) assay kit were obtained from Megazyme International Ireland Ltd. (Wicklow, Ireland). All other chemicals used in the study were analytical grade.

### 2.2 Extraction of Pineapple Stem Starch

Pineapple stems (20 kg) were processed using a wet‐milling method based on the procedures outlined by Nakthong et al. (2017) with minor modifications, to obtain pineapple stem starch (PSS). Briefly, the pineapple stems were first washed, peeled, and cut into small pieces. These pieces of peeled pineapple stems (10.25 kg) were then ground with distilled water at a 1:2 ratio using a blender (HR2221, Philips, China; under the supervision of Philips Domestic Appliances Holding B.V., the Netherlands) and filtered sequentially through 100‐, 140‐, and 270‐mesh screens to remove fibrous materials. The filtrate was centrifuged at 3000 × *g* for 10 min using a universal laboratory centrifuge (HERMLE Z 400 K, HERMLE Labortechnik GmbH, Wehingen, Germany) to separate the starch, which was repeatedly washed, centrifuged, and decanted until the supernatant became clear. Finally, the starch sediment was dried overnight at 45°C in a hot‐air oven (BINDER FD56, BINDER GmbH, Tuttlingen, Germany) and then sieved through a 100‐mesh screen to obtain a fine powder of PSS (1.10 kg). The starch yield, calculated on a wet basis relative to the mass of unpeeled and peeled pineapple stems, was 5.50% and 10.73%, respectively.

### 2.3 Safety Evaluation of Pineapple Stem Starch

Starch extracted from pineapple stems was considered a novel food ingredient; therefore, the safety of PSS was assessed based on the relevant food safety and microbiological criteria for its intended use. Lead (Pb) and cadmium (Cd) contents of PSS were determined to evaluate chemical safety. Sample was acid‐digested and analyzed using atomic absorption spectrophotometry (AAS) according to AOAC Official Method (AOAC 2019).

Microbiological analyses were performed following the established protocols for *Escherichia coli* (U.S. Food and Drug Administration 2020), *Salmonella* spp. (International Organization for Standardization 2017), and *Staphylococcus aureus* (U.S. Food and Drug Administration 2016).

Additionally, moisture content and water activity, which are relevant to microbial growth and food safety, were also determined. Moisture content of PSS was determined by oven drying (method 925.45), according to the Association of Official Analytical Chemists (AOAC 2019). Water activity was measured at 25°C using a water activity meter (Novasina AG, Lachen, Switzerland). The PSS sample was placed in a water activity cup to approximately two‐thirds of the cup before analysis (Sriprablom et al. 2022).

### 2.4 Preparation of Gluten‐Free Biscuits

In our preliminary study, the gluten‐free control biscuit recipe consisted of the following ingredients: RF (100 g), SRBO (60 g), MFS (40 g), water (25 g), whole egg (20 g), SPI (15 g), baking powder (3.0 g), CMC (1.5 g), vanilla butter flavor (1.5 g), sodium bicarbonate (1 g), and salt (1.0 g). To formulate biscuits with varying levels of PSS, the amount of RF was replaced with PSS at 25%, 50%, 75%, and 100% (w/w). These substitution levels were selected based on the experimental design previously reported for rice flour‐PSS noodles by Pinyo et al. (2024), allowing the effects of PSS incorporation to be evaluated over a wide substitution range. Other ingredients remained the same as in the control formulation, with only the amount of water varying. For the formulations containing 25%, 50%, 75%, and 100% PSS, the amount of water used was adjusted to 25, 24, 22, and 20 g, respectively, to maintain the desired dough consistency and functionality. For biscuit preparation, SRBO and MFS were first mixed and beaten using a hand mixer (EHM3407 model, Electrolux, Stockholm, Sweden) for 3–4 min until a creamy texture was achieved, after which a whole egg and vanilla butter flavor were added to the mixture. Then, the blended mixtures of RF and PSS at different ratios, along with the remaining ingredients, were added and thoroughly mixed to form a dough. The dough was kneaded and sheeted to a uniform thickness of 0.4 cm and cut into a biscuit size of 2.5 × 5 cm. Baking was carried out at 160°C–170°C for 15 min in an oven (EO‐70 K model, Sharp Corporation, Osaka, Japan). The biscuit samples were cooled to room temperature (25°C) and stored in sealed plastic bags.

### 2.5 Texture and Geometrical Measurement

Dough hardness and adhesiveness were measured using a TA‐XTplus texture analyzer (Stable Micro Systems, Surrey, UK) using an aluminum cylinder probe of 50 mm diameter (P/50). The test was conducted at a pretest speed of 1 mm/s and a test and posttest speed of 5 mm/s, applying a compression strain of 75%. Biscuit hardness was measured by a cutting force using a three‐point bend rig (HDP/3 PB) with a sharp blade probe of 9 cm long and 0.5 cm thick according to Sriprablom et al. (2022). Texture analysis was conducted with 10 replicate measurements for each batch. Width, length, and thickness of baked biscuits were measured using a Vernier caliper at three different points.

### 2.6 Color Measurement

The surface color of the biscuit samples was measured in the L*, a*, b* system using a ColorFlex EZ spectrophotometer (Hunter Associates Laboratory Inc., Virginia, USA), according to the method described by Sriprablom et al. (2022). The ingredient composition of the biscuits may influence the Maillard reaction during baking, thereby affecting biscuit color and resulting in lighter or darker products. Additionally, factors such as moisture content of the dough may also influence color changes. The biscuit samples were placed into a glass sample cup with the surface of biscuits facing the bottom. The sample cup was then positioned at the sample port with the side to be measured facing the port, and covered with a sample cup cover. In this color system, L* represents the lightness, and a* and b* are the color coordinates where +a* is the red, −a* is the green, +b* is the yellow, and −b* is the blue directions.

### 2.7 In Vitro Starch Digestibility of Biscuits

The digestibility of starch in the gluten‐free biscuits was determined according to the procedures of Englyst et al. (1992) with slight modifications. The ground biscuits (0.80 g, dry basis), guar gum (0.05 g), and pepsin‐HCL solution (10 mL) were mixed thoroughly. Then, the mixture was placed in a water bath at 37°C with a stirring speed of 160 rpm for 30 min to digest the protein. After that, 10 mL of sodium acetate buffer (0.25 M, pH 5.2) was added and mixed with an enzyme solution of pancreatin and amyloglucosidase (5 mL). The mixture was incubated at 37°C in a water bath with shaking for 2 h. During incubation, aliquots (0.25 mL) of the mixture were taken at different time points (20, 60, 90, and 120 min). These aliquots were then mixed with ethanol (66% v/v, 10 mL) to terminate the enzymatic reaction and then centrifuged at 1500 × *g* for 10 min. The supernatant was used to analyze the amount of glucose released using the GOPOD assay kit. The percentage of starch hydrolysis at different hydrolysis times and starch fractions were calculated as follows:

$$\text{Starch hydrolysis in each hydrolysis time}\;\left(\%\right)=\left(G_{t}/\mathrm{TS}\right)\times 0.9\times 100$$

$$\text{Rapidly digestible starch}\;\left(\mathrm{RDS}\right)\;\left(\%\right)=\left(G20/\mathrm{TS}\right)\times 0.9\times 100$$

$$\text{Slowly digestible starch}\;\left(\mathrm{SDS}\right)\;\left(\%\right)=\left[\left(G120–G20\right)/\mathrm{TS}\right]\times 0.9\times 100$$

$$\text{Resistant starch}\;\left(\mathrm{RS}\right)\;\left(\%\right)=100–\left(\mathrm{RDS}+\mathrm{SDS}\right)$$

where *G*_*t*_ is the glucose content released at each hydrolysis time point; G20 and G120 are the glucose content released at 20 and 120 min of hydrolysis, respectively; TS is total starch content, determined using a total starch assay kit.

### 2.8 Estimated Glycemic Index (eGI) of Biscuits

The eGI was calculated from the hydrolysis index (HI) value of each sample according to the method recommended by Goñi et al. (1997). The starch hydrolysis rate was expressed as the percentage of TS hydrolyzed at each time interval (0, 20, 60, 90, and 120 min). The area under the hydrolysis curve (AUC) was calculated using the nonlinear first‐order equation to describe the kinetics of hydrolysis of the samples:

$$\mathrm{AUC}=C_{\infty }\left(t_{f}-t_{o}\right)-\left(C_{\infty }/k\right)\;\left[1–\exp\;\left[-k\left(t_{f}-t_{o}\right)\right]\right]$$

where *C*_∞_ corresponds to the equilibrium percentage of starch hydrolyzed after 120 min, *t*_*f*_ is the final time (120 min), *t*_o_ is the initial time (0 min) and *k* is the kinetic constant.

The HI value was obtained by dividing the AUC of a sample by the corresponding AUC of white bread (a reference food) and expressing the result as a percentage (Cairano et al. 2021; Goñi et al. 1997; Naseer et al. 2021). The eGI was calculated using the following equation:

eGI=39.71+0.549×HI

### 2.9 Sensory Evaluation

The Mahidol University Central Institutional Review Board has approved all protocols (Protocol Number 2024/244.0506), and the panelists have given their written consent prior to participation. A 9‐point hedonic scale was selected for sensory evaluation because it offers a reliable and widely accepted method for assessing consumer preferences and overall acceptability of food products. Participants were eligible for inclusion if they were Mahidol University students or staff members aged 18 years or older, regardless of gender, non‐smokers, and had no conditions affecting sensory perception (e.g., color blindness, chronic rhinitis, or oral/throat diseases). Individuals were excluded if they had a history of allergies to any of the biscuit ingredients (rice flour, pineapple stem starch, shortening rice bran oil, and monk fruit sweetener) or expressed a dislike for biscuits. Individuals who did not meet the inclusion criteria or declined to participate were not enrolled in the study.

The five gluten‐free biscuit samples were individually presented in sealed containers (one biscuit per container) and labeled with random 3‐digit codes. The samples were evaluated by 30 untrained panelists recruited from the university community (12 males and 18 females; mean age = 23.6 ± 3.0 years). A minimum of 30 panelists is recommended for hedonic testing to ensure reliable sensory evaluation (Curtis 2013). Each panelist evaluated five biscuit samples, which were presented in a randomized order to minimize presentation bias. Drinking water was provided, and panelists were instructed to rinse their mouths between samples to reduce carry‐over effects. The panelists were asked to assign scores using a 9‐point hedonic scale ranging from 1 (dislike extremely) to 9 (like extremely) for appearance, color, flavor, texture, and overall acceptability. Sensory evaluation data were analyzed using one‐way analysis of variance (ANOVA), followed by Tukey's multiple comparison test to determine significant differences among the biscuit formulations at a significance level of *p* ≤ 0.05 (Sharma et al. 2016).

### 2.10 Proximate Analysis

Proximate analysis of gluten‐free control biscuits and optimal PSS biscuits was performed according to the Association of Official Analytical Chemists (AOAC 2019) method. Moisture content was determined by oven drying (method 925.45), crude protein content by Kjeldahl method (method 991.20), total fat content by acid hydrolysis and solvent extraction using Soxtec (method 922.06), dietary fiber content by Prosky method (method 985.29), and ash content by dry ashing (method 930.30). Total carbohydrate content was calculated by subtracting the percentages of moisture, protein, fat, and ash contents from 100%. Energy was calculated as the sum of kilocalories from carbohydrate, protein, and fat according to Atwater system (Merrill and Watt 1973) as follows:

Energy (kcal) = (4 × carbohydrate) + (4 × protein) + (9 × fat).

### 2.11 Storage Stability

Gluten‐free control biscuits and optimal PSS biscuits (25 g) were packaged with aluminum foil bag and stored under accelerated conditions at 45°C for 2 months. According to the calculations based on the Arrhenius equation, assuming a Q_10_ value of 2 for the deteriorative reaction (Chanadang and Chambers 2019; Radoš et al. 2022), storage at 45°C for 60 days (2 months) is equivalent to approximately 8 months of storage at 25°C. Therefore, this accelerated storage period modeled the typical expected shelf‐life of commercial low‐moisture biscuits. The lipid oxidation rate was monitored by measuring the peroxide value (PV) and the thiobarbituric acid reactive substances (TBARS) of the oil extracted from the biscuits. The extraction of lipids from biscuits for lipid oxidation analysis was performed according to the method of Al‐Bandak and Oreopoulou (2011). PV was determined following the procedures described by Al‐Bandak and Oreopoulou (2011) and TBARS was measured using the method of Supawong et al. (2019) to evaluate storage stability of biscuits on Days 0, 20, 40, and 60. The PV results were expressed as meq of peroxide/kg of oil, and the TBARS results were expressed as mg malonaldehyde/kg of oil.

### 2.12 Statistical Analysis

Two independent baking batches were prepared for each biscuit formulation. All analytical measurements were conducted in at least triplicate for each batch, unless otherwise stated. Results are expressed as means and standard deviations. The statistical analyses were performed using SPSS version 21.0 (SPSS Inc., Illinois, USA). The one‐way ANOVA test with Tukey's post hoc test and independent‐samples *t*‐test (only for comparison of chemical composition between gluten‐free control biscuits and optimal PSS biscuits) were used to determine the statistically significant differences (*p* ≤ 0.05).

## 3 Results and Discussion

### 3.1 Safety Assessment of Pineapple Stem Starch

Prior to the formulation of biscuits, a comprehensive safety assessment of PSS was conducted. The safety evaluation of raw materials is a fundamental requirement for ensuring the quality and consumer acceptance of novel food products. In this study, the evaluation focused on two primary areas: heavy metal contamination and microbiological quality (Table 1). The heavy metal analysis revealed that both lead (Pb) and cadmium (Cd) were not detected in the starch samples. The absence of these contaminants indicates that the raw materials and production conditions were adequately controlled, minimizing potential risks associated with environmental contamination. Only negligible concentrations of heavy metals were found in the starch extracted from pineapple stem (Rinju and Harikumaran‐Thampi 2021).

**TABLE 1:** Safety assessment of pineapple stem starch.

Parameters	Unit	Results
Heavy metals		
Lead (Pb)	mg/kg	Not detected
Cadmium (Cd)	mg/kg	Not detected
Microbiological quality		
*Escherichia coli*	MPN/g	< 3
*Salmonella* spp.	per 25 g	Not detected
*Staphylococcus aureus*	per 0.1 g	Not detected
Moisture content	%	9.96 ± 0.15
Water activity	—	0.27 ± 0.01

Regarding microbiological quality (Table 1), *Escherichia coli* levels were below 3 MPN/g, indicating satisfactory hygienic conditions. Moreover, pathogenic microorganisms, including *Salmonella* spp. and *Staphylococcus aureus* , were not detected in 25 g and 0.1 g samples, respectively. These results demonstrate that PSS complies with the microbiological quality criteria for foods according to the Notification of the Ministry of Public Health (No. 416) B.E. 2563 (2020) (Ministry of Public Health 2020). This safety assessment provides a robust foundation for the incorporation of PSS into food formulations and supports its potential as a sustainable alternative food source. Moreover, the extracted PSS had a moisture content of 9.96% and a very low water activity (0.27) (Table 1), which is below 0.6, the minimum water activity generally required for microbial growth (Rutkowska et al. 2023). These finding indicated that PSS had low susceptibility to microbial growth and was stable under low‐moisture condition.

The antinutritional factors in PSS were not quantified in this study, as previous literature indicated that pineapple by‐products contain only low levels of antinutrients that do not pose significant health risks (Emmanuella et al. 2025; Onyenweaku and Kesa 2024; Soni et al. 2023). Furthermore, the wet‐milling extraction method used to prepare PSS involves repetitive aqueous washing, which yields a relatively purified starch fraction and may facilitate the removal of water‐soluble antinutritional components. Consequently, residual antinutrient levels in PSS are expected to be negligible. Nevertheless, the absence of direct antinutrient quantification represents a limitation of the current study and warrants further investigation.

### 3.2 Geometrical Properties of Biscuits

According to Table 2, the results for width, length, and thickness of biscuits varied from 2.51 to 2.97 cm, 5.03 to 5.48 cm, and 0.55 to 0.77 cm, respectively. Higher PSS levels resulted in greater physical dimensions compared to the control biscuits made with 100% RF. This result was aligned with the visual appearance and cross‐section of gluten‐free biscuits, which showed increased thickness with higher PSS levels (Figure 1). The protein in RF may help stabilize dough structure. Replacing RF with higher PSS ratios, along with increased starch content but lower protein content, may limit dough structural forming and contribute to weak structural stability. Consequently, this led to a softer and stickier dough, as indicated by significantly lower dough hardness and higher dough adhesiveness in Table 2. The weaker and stickier dough structure enhanced the dough rise during baking, leading to greater biscuit dimensions (width, length, and thickness). It was reported that the thickness of gluten‐free biscuits was negatively correlated with dough hardness (*r* = −0.884) but positively correlated with dough stickiness (*r* = 0.755) (Schober et al. 2003).

**TABLE 2:** Physical and color characteristics of gluten‐free biscuits made from blends of all‐purpose rice flour (RF) and pineapple stem starch (PSS) at different ratios.

Parameter	RF:PSS ratio				
	100:0	75:25	50:50	25:75	0:100
Width (cm)	2.51 ± 0.01^e^	2.57 ± 0.03^d^	2.68 ± 0.03^c^	2.82 ± 0.03^b^	2.97 ± 0.03^a^
Length (cm)	5.03 ± 0.06^e^	5.13 ± 0.06^d^	5.28 ± 0.03^c^	5.39 ± 0.02^b^	5.48 ± 0.03^a^
Thickness (cm)	0.55 ± 0.01^e^	0.61 ± 0.01^d^	0.65 ± 0.01^c^	0.69 ± 0.01^b^	0.77 ± 0.01^a^
Dough hardness (N)	311.66 ± 9.58^a^	266.40 ± 10.68^b^	244.80 ± 13.49^c^	231.29 ± 12.05^c^	195.46 ± 11.31^d^
Dough adhesiveness (N.s)	1.11 ± 0.10^a^	1.51 ± 0.32^b^	2.08 ± 0.11^c^	2.32 ± 0.32^c^	3.04 ± 0.45^d^
Biscuit hardness (N)	8.41 ± 0.37^a^	8.01 ± 0.33^b^	5.32 ± 0.33^c^	5.12 ± 0.35^d^	4.75 ± 0.18^e^
L*	60.80 ± 0.44^a^	61.19 ± 0.48^a^	61.20 ± 0.17^a^	61.23 ± 0.11^a^	61.24 ± 0.07^a^
a*	12.92 ± 0.07^a^	12.82 ± 0.17^a^	12.59 ± 0.22^b^	12.57 ± 0.18^b^	12.56 ± 0.20^b^
b*	33.21 ± 0.59^a^	33.59 ± 0.32^a^	33.69 ± 0.04^a^	33.71 ± 0.23^a^	33.77 ± 0.34^a^

*Note:* Mean ± standard deviation values of two independent baking batches (*n* = 2) followed by different superscript letters within the same row are significantly different (*p* ≤ 0.05). (*) represents the color parameters L*, a*, and b*.

**FIGURE 1:**
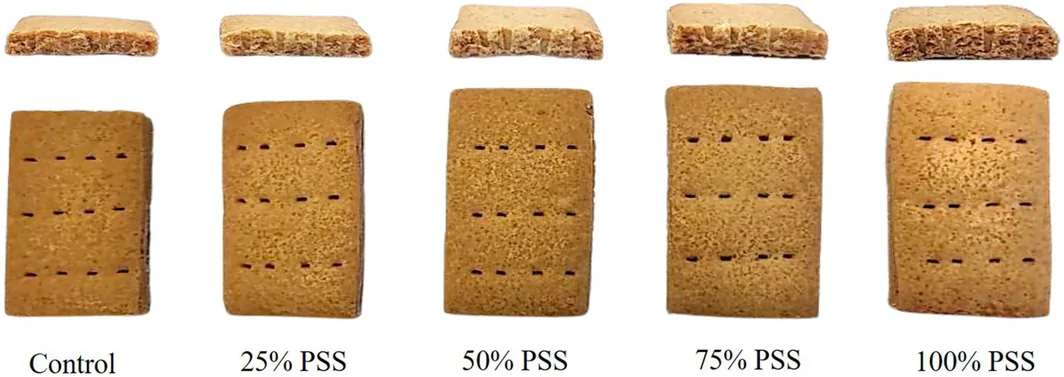
Visual appearance of gluten‐free biscuits made from blends of all‐purpose rice flour (RF) and pineapple stem starch (PSS) at different ratios.

### 3.3 Textural Properties of Dough and Biscuits

As the substitution levels of PSS increased, the dough hardness decreased but the dough adhesiveness increased, consequently leading to a decreased hardness of biscuits, compared to 100% RF biscuits (Table 2). Increased PSS levels improved the rising capacity of biscuits, contributing to a softer texture of biscuits, a desirable characteristic for consumers. Previous studies reported that the firmness of gluten‐free biscuits had a significant positive correlation with dough hardness (*r* = 0.881, *p* ≤ 0.05) and a significant negative correlation with dough stickiness (*r* = −0.964, *p* ≤ 0.01) (Schober et al. 2003). These findings were consistent with the results of Bolarinwa et al. (2019), who reported that the dough and biscuit hardness were lower in the rice‐potato starch biscuits compared to 100% rice flour biscuits. Moreover, RS might also affect the product's hardness. A higher RS level provides an increased fiber content and water‐binding capacity, which contributes to a reduction in biscuit hardness (Jiamjariyatam et al. 2024).

### 3.4 Color Characteristics of Biscuits

The surface color analysis of all biscuits revealed L*, a* and b* values ranging from 60.80 to 61.24, 12.56 to 12.92, and 33.21 to 33.77, respectively (Table 2). The redness of the biscuits slightly decreased with an increase of PSS ratio. However, there was no significant difference (*p* > 0.05) for L* and b* parameters (Table 2). The results indicated that the addition of PSS had little effect on the surface color of gluten‐free biscuits in this study. This finding aligned with the report by Mancebo et al. (2016), which noted that the color of gluten‐free rice flour biscuits was unaffected by the addition of maize starch.

### 3.5 In Vitro Starch Digestibility of Biscuits

The results of starch hydrolysis rate of gluten‐free biscuits are shown in Figure 2. After 2 h hydrolysis, the biscuits with 100% PSS had the lowest starch digestion rate (36.62%), while the control (100% RF) biscuits had the highest rate (88.30%). This finding demonstrated that the starch hydrolysis rate of gluten‐free biscuits decreased as the proportion of PSS increased. Similar results were reported for gluten‐free biscuits prepared by incorporating a novel resistant starch (RS) ingredient derived from annealed white sorghum starch (RSWS) at different levels (15%, 30%, and 45%). The extent of in vitro starch digestion over 120 min of incubation decreased as the RSWS level increased in the formulation (Cervini et al. 2021). This suggested that the PSS could serve as an alternative source of RS in the gluten‐free biscuits, which were hydrolyzed considerably more slowly and less vulnerable to digestive enzymes compared to the 100% RF biscuits.

**FIGURE 2:**
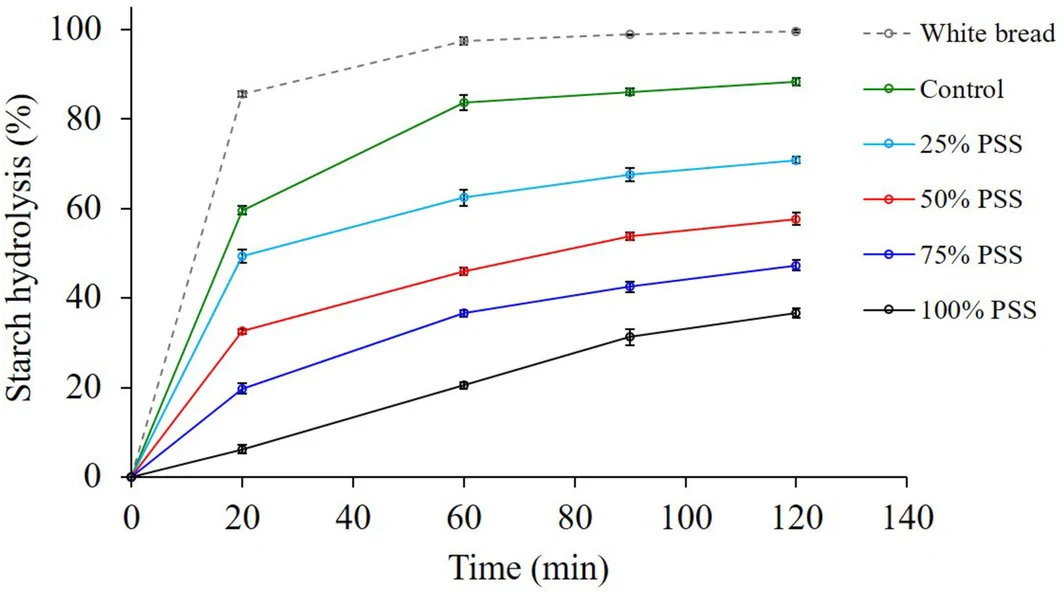
The rate of starch hydrolysis in gluten‐free biscuits made from blends of all‐purpose rice flour (RF) and pineapple stem starch (PSS) at different ratios and white bread (a reference food).

Table 3 displays the starch fractions of biscuits in terms of RDS, SDS, and RS based on the rate of starch digestion. As the proportion of PSS increased, RDS content significantly decreased, while RS content increased. In particular, the biscuits made from 100% PSS exhibited the lowest RDS content and the highest RS content (6.25% and 63.38%, respectively), corresponding with the results of starch hydrolysis rate (Figure 2). This can be attributed to the high SDS and RS content of PSS itself, which relates to its high amylose content (30.56%–30.82%) compared to commercial rice flour, as reported by Pinyo et al. (2024) and Sriprablom et al. (2023). The higher the amylose content in starch, the more it resists enzymatic digestion by limiting starch swelling (Sriprablom et al. 2023). Therefore, PSS was a good source for improving the RS content of food products without requiring any modification. The total starch content of biscuits increased as the proportion of PSS substitution rose. A similar result was found in rice flour noodles when substituted with PSS (Pinyo et al. 2024).

**TABLE 3:** In vitro starch digestibility and estimated glycemic index (eGI) of gluten‐free biscuits made from blends of all‐purpose rice flour (RF) and pineapple stem starch (PSS) at different ratios.

RF:PSS ratio	TS (%)	RDS (%)	SDS (%)	RS (%)	*k*	HI	eGI
White bread	67.43 ± 0.74^a^	85.62 ± 0.65^a^	13.99 ± 0.34^e^	0.39 ± 0.35^f^	0.0739 ± 0.00^a^	100.00 ± 0.00^a^	94.61 ± 0.00^a^
100:0	41.14 ± 0.66^e^	59.58 ± 1.01^b^	28.72 ± 1.59^b^	11.70 ± 0.90^e^	0.0500 ± 0.01^b^	82.74 ± 1.08^b^	85.13 ± 0.59^b^
75:25	42.04 ± 0.73^de^	49.37 ± 1.56^c^	21.44 ± 0.88^d^	29.19 ± 0.69^d^	0.0436 ± 0.00^b^	64.84 ± 0.21^c^	75.30 ± 0.11^c^
50:50	43.85 ± 1.18^cd^	32.54 ± 0.52^d^	25.11 ± 1.29^c^	42.35 ± 1.35^c^	0.0333 ± 0.00^c^	48.61 ± 0.33^d^	66.40 ± 0.18^d^
25:75	45.43 ± 0.33^c^	19.76 ± 1.16^e^	27.53 ± 1.30^bc^	52.17 ± 1.14^b^	0.0259 ± 0.00^d^	37.03 ± 0.56^e^	60.04 ± 0.31^e^
0:100	47.25 ± 0.57^b^	6.25 ± 0.92^f^	30.36 ± 1.73^a^	63.38 ± 1.01^a^	0.0149 ± 0.00^e^	22.97 ± 0.78^f^	52.32 ± 0.43^f^

*Note:* Mean ± standard deviation values of two independent baking batches (*n* = 2) followed by different superscript letters within the same column are significantly different (*p* ≤ 0.05).

Abbreviations: HI, hydrolysis index; *k*, kinetic constant; RDS, rapidly digestible starch; RS, resistant starch; SDS, slowly digestible starch; TS, total starch.

### 3.6 Estimated Glycemic Index of Biscuits

In addition, other parameters of starch digestibility, that is, kinetic constant (*k*), hydrolysis index (HI), and estimated glycemic index (eGI) are also presented in Table 3. To determine the HI and eGI, fresh white wheat bread was used as a reference food (Goñi et al. 1997). As the PSS ratio increased, the *k* value decreased, resulting in a reduction in HI and subsequently lower eGI value. The lower *k* value suggests generally higher resistance to enzymatic hydrolysis (Barine and Yorte 2016).

Control biscuits made from 100% RF exhibited the highest HI and eGI values (82.74% and 85.13%, respectively) (Table 3), indicating that the starch was hydrolyzed quickly and may potentially raise blood sugar level upon consumption. Whereas the HI and eGI values of biscuits incorporated with PSS were significantly decreased (64.84%–22.97% HI and 75.30R–52.30% GI) with increasing PSS ratio, which may be attributed to low RDS, high SDS and/or RS (Agama‐Acevedo et al. 2012; Chinma et al. 2022). Foods can be classified into three main categories based on their GI values: low GI (≤ 55), medium GI (56–69), and high GI (≥ 70) (Atkinson et al. 2021). In this study, the eGI of the 100% PSS biscuits was 52.32, which can be regarded as low GI products. The gluten‐free biscuits incorporated with 50%–75% PSS showed eGI values in the range of 60.04–66.40, classifying them as medium GI products. The GI concept enabled the classification of various carbohydrate‐rich foods based on their impact on glycemic levels after meals (Giuberti et al. 2017). Currently, there is growing interest in reducing the GI of highly digestible foods, as consuming lower‐GI foods over time may positively impact insulin and postprandial responses, and could aid in preventing and managing obesity, diabetes, hyperlipidemia, and chronic metabolic diseases (Cairano et al. 2021; Chinma et al. 2022; Giuberti et al. 2017).

### 3.7 Sensory Evaluation of Biscuits

Sensory attributes of the biscuits were evaluated using a nine‐point hedonic scale, as shown in Table 4. Regarding sensory qualities, the results showed no significant differences in color (*p* > 0.05) between biscuits containing 25%–100% PSS and the control biscuits (100% RF). The control biscuits received the lowest preference scores in appearance, flavor, texture, and overall acceptability, likely due to their oily surface and hard texture. The compact structure and high hardness of control biscuits (Figure 1 and Table 2) likely hindered the uniform distribution of SRBO within the biscuits, resulting in an oily surface observed during storage. Moreover, as the proportion of PSS rose from 25% to 75%, the consumer preference scores for gluten‐free biscuits significantly increased. The highest scores for all quality attributes were found in biscuits containing 75% PSS. These biscuits achieved an overall acceptability score of 6.83, ranging from “slight liking” to “moderate liking,” possibly due to their high rising ability and reduced hardness (Table 2). Conversely, substitution of RF with 100% PSS resulted in significantly lower hedonic scores for most sensory attributes, leading to a significant decrease in overall acceptability (*p* ≤ 0.05). The 100% PSS biscuits had a soft, crumbly texture due to their weak structure, which may be attributed to their high starch and low protein content, resulting in significantly lower consumer preference scores than those of the biscuits containing 75% PSS (Table 4). However, all formulations received overall acceptability scores above the limit of acceptability (i.e., 5) (Cervini et al. 2021), indicating they can be considered good‐quality products. As a result, the best formulation of gluten‐free biscuits, which had a medium eGI (60.04) and the highest overall acceptability score (6.83) was a blend of 25% RF and 75% PSS.

**TABLE 4:** Sensory evaluation of gluten‐free biscuits made from blends of all‐purpose rice flour (RF) and pineapple stem starch (PSS) at different ratios.

RF:PSS ratio	Appearance	Color	Flavor	Texture	Overall acceptability
100:0	5.90 ± 1.47^b^	6.83 ± 1.12^a^	5.77 ± 1.36^b^	5.17 ± 1.29^c^	5.73 ± 1.01^c^
75:25	6.07 ± 1.48^b^	6.87 ± 1.11^a^	6.20 ± 1.32^ab^	5.97 ± 1.20^bc^	6.30 ± 0.89^b^
50:50	6.07 ± 1.01^b^	6.80 ± 0.71^a^	6.50 ± 1.14^a^	6.47 ± 1.50^a^	6.57 ± 1.18^ab^
25:75	7.03 ± 1.31^a^	7.00 ± 1.17^a^	6.53 ± 1.20^a^	6.53 ± 1.48^a^	6.83 ± 1.23^a^
0:100	6.20 ± 1.19^b^	6.50 ± 1.04^a^	6.40 ± 1.43^a^	6.13 ± 1.14^b^	6.60 ± 1.28^ab^

*Note:* Mean ± standard deviation values (*n* = 30) followed by different superscript letters within the same column are significantly different (*p* ≤ 0.05).

### 3.8 Proximate Composition of Biscuits

In this study, gluten‐free biscuits made from RF‐PSS blends with a 25:75 ratio were selected for proximate composition analysis, compared with 100% RF (control), due to receiving the highest panelist acceptance scores (Table 4), as shown in Table 5. The results showed that moisture, protein, available carbohydrate, and dietary fiber between the control and the biscuits containing 75% PSS were significantly different (*p* ≤ 0.05). The moisture content of the biscuits containing 75% PSS (1.05%) was significantly lower than that of control biscuits (2.75%). The weaker structure and higher rising ability of the 75% PSS formulation likely facilitated water evaporation during baking, reducing the final moisture content. As a result, less water (22 g) was added to the 75% PSS formulation to achieve a desirable dough consistency. The lower initial water content in the formulation may also explain the lower moisture content in the 75% PSS biscuits after baking. Biscuits typically have very low moisture content, ranging from 1% to 5%, which plays a crucial role in preventing microbiological spoilage, and thus extending shelf life (Ng et al. 2017; Ogunbusola et al. 2020).

**TABLE 5:** Chemical composition and energy value of gluten‐free biscuits made from blends of all‐purpose rice flour (RF) and pineapple stem starch (PSS) at different ratios.

RF:PSS ratio	Moisture (%)	Protein (%)	Total fat (%)	Ash (%)	Dietary fiber (%)	Total carbohydrate (%)	Energy (kcal/100 g)
100:0	2.75 ± 0.01^a^	10.93 ± 0.01^a^	27.90 ± 0.01^a^	1.98 ± 0.02^a^	0.83 ± 0.02^b^	56.45 ± 0.04^b^	520.60 ± 0.07^b^
25:75	1.05 ± 0.02^b^	8.34 ± 0.01^b^	27.66 ± 0.23^a^	1.97 ± 0.01^a^	1.65 ± 0.04^a^	60.99 ± 0.25^a^	526.20 ± 1.05^a^

*Note:* Mean ± standard deviation values of two independent baking batches (*n* = 2) followed by different superscript letters within the same column are significantly different (*p* ≤ 0.05).

Furthermore, the substitution of RF with 75% PSS in biscuits resulted in a decrease in protein content. This could be due to the difference in protein content between the raw RF and PSS (data not shown) used in the biscuit preparation in this study. In general, PSS consists of very low protein content (0.71%) (Nakthong et al. 2017) compared to Thai rice flour (6.3%–10.7%) (Leewatchararongjaroen and Anuntagool 2016). Furthermore, the protein content of both biscuit samples was significantly high due to the addition of other sources of protein, such as soy protein isolate and eggs, in the gluten‐free biscuit formulation. On the other hand, the replacement of RF with 75% PSS caused a significant increase in total carbohydrate and dietary fiber content. It was due to the fact that 75% PSS biscuits have a high level of RS content (Table 3), resulting in higher dietary fiber content compared to 100% RF biscuits. An increase in total dietary fiber content has been reported in gluten‐free biscuits made by partial replacement of commercial rice flour with RS ingredients from modified waxy rice starch (Giuberti et al. 2017). However, the total fat and ash contents did not show any significant differences (*p* > 0.05) between the two biscuits. Similar results have been reported for gluten‐free rice biscuits made from rice flour with different amylose contents and cowpea beans (Dierings de Souza et al. 2021), or in combination with RS sources from modified waxy rice starch (Giuberti et al. 2017). The energy values for both biscuits (521–526 kcal/100 g) did not differ substantially. This study primarily focused on the proximate chemical compositions (macronutrients) in the developed gluten‐free biscuits. However, the lack of micronutrients (vitamins and minerals) analyses was acknowledged as a limitation of the study, and should be further investigated to provide the comprehensive information on nutritional properties of the developed biscuits.

### 3.9 Storage Stability (PV and TBARS) of Biscuits

According to the highest sensory evaluation scores, the 75% PSS biscuits were selected for storage stability study, in comparison to the control biscuits (100% RF). Due to their high lipid content (Table 5), the PV and TBARS values of both gluten‐free biscuits were measured under accelerated storage conditions (Figure 3). In this study, the SRBO used in preparation of healthy gluten‐free biscuits contains a high level of unsaturated fatty acids (Kaur et al. 2012), which makes the biscuits susceptible to lipid oxidation and rancidity during storage. PV is a measurement of hydroperoxide content formed during the initial stages of lipid oxidation. While TBARS value measures the formation of secondary oxidation products (mainly malondialdehyde), resulting from the breakdown of primary oxidative products, which is a direct cause of rancidity and off‐odors (Bialek et al. 2016; Gogoi et al. 2020). Higher PV and TBARS values indicate greater production of hydroperoxides and a higher level of oxidation. The results showed that the control biscuits and biscuits containing 75% PSS were not different in PV and TBARS values and showed little changes during the first 20 days of storage (Figure 3). However, after 20 to 60 days of storage, PV and TBARS values increased significantly for both types of biscuits, with 75% PSS biscuits showing higher values than the control. This may be due to the lower presence of bioactive antioxidant compounds in the PSS, while rice flour contains phenolics with antioxidant properties (Pereira et al. 2021). Therefore, these bioactive compounds can more effectively delay lipid oxidation in 100% RF biscuits compared to biscuits containing 75% PSS during storage. Nevertheless, both control and 75% PSS biscuits exhibited PV within the permissible limit of 10–20 meq of peroxide/kg of fat (Hashem et al. 2023; Jeyasanta et al. 2013) throughout the storage period, with values of 7.17 and 10.50 meq of peroxide/kg of fat for control and 75% PSS biscuits, respectively, at 60 days of storage. In addition, the TBARS values of both biscuits were found to be below the acceptable limit, which is < 3 mg MDA/kg (Hashem et al. 2023; Jeyasanta et al. 2013). The results showed that the control biscuits had a TBARS value of 2.03 mg MDA/kg of fat, while the 75% PSS biscuits had a value of 2.73 mg MDA/kg of fat, indicating good quality in both biscuit products. Despite its high levels of unsaturated fatty acids, SRBO contains natural antioxidants such as tocopherols, tocotrienols, and oryzanols, which provide excellent resistance to rancidity and oxidative deterioration, eventually extending the shelf life of food products (Sharif et al. 2003).

**FIGURE 3:**
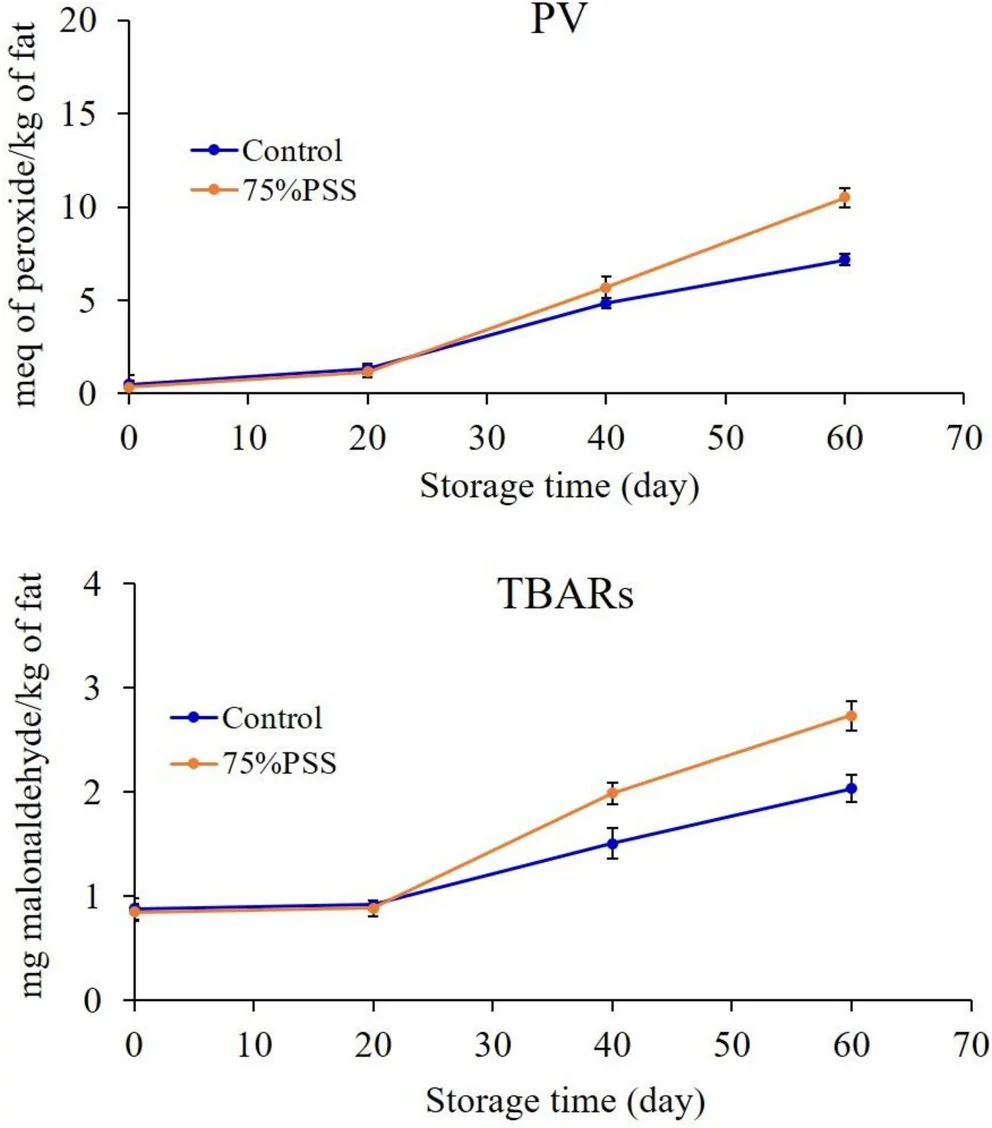
Changes in the PV and TBARs contents of gluten‐free rice biscuits with 75% PSS and without PSS (control) during 60 days of storage.

## 4 Conclusions

This study demonstrates that PSS is safe for functional food applications, as it complies with relevant heavy metal and microbiological safety standards for human consumption. Substituting rice flour (RF) with pineapple stem starch (PSS) significantly influenced the properties of gluten‐free biscuits. Biscuits containing PSS exhibited higher thickness than gluten‐free control biscuits made with 100% RF, suggesting a softer texture. Higher PSS levels were associated with increased resistant starch content and a lower estimated glycemic index (eGI). The biscuits with a 25:75 RF‐to‐PSS ratio achieved the highest liking scores across all evaluated sensory attributes and exhibited a medium glycemic index (eGI = 60.04). These optimal biscuits (75% PSS) also had higher dietary fiber content than the control and demonstrated good storage stability. The findings highlight the potential for commercially developing reduced‐GI, high‐quality gluten‐free biscuits using RF‐PSS blends. However, further animal and human studies are needed to confirm the safety and health benefits of PSS‐containing biscuits. Moreover, microbiological analyses should also be included in the storage stability studies to comprehensively assess product safety during storage. Incorporating PSS into food products not only has the potential to improve health outcomes but also aids in minimizing agricultural waste.

## Author Contributions

**Jiratthitikan Sriprablom:** conceptualization, methodology, investigation, formal analysis, validation, writing – original draft. **Phenphop Phansuea:** conceptualization, methodology, investigation, formal analysis, validation, writing – original draft, funding acquisition. **Yuraporn Sahasakul:** investigation, formal analysis, writing – review and editing. **Sasapin Disnil:** investigation, validation. **Amornrat Aursalung:** investigation, validation. **Nuttarat Srisangwan:** investigation, validation. **Arisa Keeratichamroen:** investigation, validation. **Supatcha Srisungwan:** investigation, validation. **Rungtiwa Wongsagonsup:** conceptualization, supervision, methodology, writing – review and editing.

## Ethics Statement

This study involved sensory evaluation of gluten‐free biscuits developed from pineapple stem starch. All study protocols were approved by Mahidol University Central Institutional Review Board (Protocol Number 2024/244.0506). Written informed consent was obtained from all panelists prior to participation.

## Conflicts of Interest

The authors declare no conflicts of interest.

## Data Availability

The data that support the findings of this study are available from the corresponding author upon reasonable request.
